# Novel Hierarchical Disordered Li- and Al-KIL-2 Catalysts for the Pyrolysis of Biomass Model Compounds and Wool Waste: A Comparison with ZSM-5

**DOI:** 10.3390/molecules29235719

**Published:** 2024-12-04

**Authors:** Roozbeh Kalateh, Tevfik Aysu, Manuel Ojeda, Aimaro Sanna

**Affiliations:** Institute of Mechanical, Process and Energy Engineering, School of Engineering and Physical Sciences, Heriot-Watt University, Edinburgh EH12 7NT, UK; roozbehkalateh@outlook.com (R.K.); tevfikaysu@hotmail.com (T.A.); m.ojeda@hw.ac.uk (M.O.)

**Keywords:** thermochemical conversion, catalytic pyrolysis, lignocellulose, KIL-2, wool waste, aromatics

## Abstract

In this study, we investigated the pyrolysis of cellulose, lignin, phenylalanine and textile wool waste using microscale thermogravimetric analysis (TGA) and a gram-scale fixed bed reactor. The pyrolysis was conducted at 500 °C and 1 bar N_2_, using Al- and Li-doped mesoporous KIL-2 and ZSM-5 catalysts for comparison. Our results show that amorphous Al-KIL-2 catalyst was the most efficient in producing aromatics from cellulose and lignin. This efficiency is attributed to Al-KIL-2 large mesoporosity, wide pore size distribution, and mild acid sites. Additionally, Al-KIL-2 promoted esterification and denitrogenation reactions, indicating its potential application in the pyrolysis of biomass and protein-rich feedstocks. Conversely, the Li-KIL-2 catalyst demonstrated activity primarily in the depolymerisation of cellulose to sugars and promoted ketonisation and alcohol formation. In summary, our findings indicate that Al-KIL-2 is a promising catalyst for efficient aromatic production from biomass.

## 1. Introduction

The increasing demand for sustainable and renewable energy sources has sparked interest in the production of aromatic compounds from biomass [1,2]. Aromatics, which include compounds like benzene, toluene, and xylenes, are integral to the chemical industry, serving as precursors for a wide range of products, including plastics, pharmaceuticals, and agrochemicals. Second-/third-generation biofuels, derived from lignocellulosic biomass and waste, are expected to be the primary replacements for aromatics [3]. Thermo-chemical conversion techniques, such as pyrolysis and gasification, break down biomass at high temperatures, respectively, in the absence or limited presence of oxygen. Pyrolysis generates bio-oil, which contains a complex mixture of chemical functionalities. Bio-oil is often unstable for conventional storage processes due to its high oxygen content and unsaturated compounds, such as aldehydes and ketones, which make it challenging to integrate into conventional facilities [4,5]. The bio-oil can be further refined and upgraded to enhance its aromatic content via in situ and ex situ pathways. Pre-treatment such as roasting can also generate monocyclic aromatics from biomass [6]. Bio-oil upgrading after its production is typically carried out by hydrotreating, which leads to high hydrocarbon recovery [2,7]. Catalytic cracking and hydropyrolysis have been proposed instead as in situ methods to produce aromatics. Hydropyrolysis has been shown to be highly selective for BTX, achieving a 67% yield during production [8]. Catalysts (such as zeolites) are also used for the in situ upgrading of bio-oil [6]. Zeolites are favourable catalyst materials due to their shape selectivity and acidic properties. ZSM-5, a zeolite with 10-membered rings and a three-dimensional structure, combined with micro pore sizes, is an excellent performer when it comes to producing aromatics. The specific ring structure of ZSM-5 can also prevent unfavourable side reactions, leading to higher aromatic production [9,10,11,12,13,14]. Increasing the silicon to aluminium ratio in zeolites has been found to increase the BET surface area and crystal size, and thus acidity. Bronsted acid sites are formed by aluminium atoms connected to silicon, and they are believed to be the main catalytic centre. The acidity of a zeolite is therefore related to its aluminium content, with a linear increase in overall acidity with increasing aluminium content [15].

Even though catalysts such as ZSM-5 have been intensely investigated for the catalytic pyrolysis of biomass, microporous zeolite can have limitations when it comes to converting large molecules due to the size of the pores. Therefore, there is increased interest in mesoporous materials to improve the diffusion of reactant molecules to the active sites of the catalysts. Recently, mesoporous silicates such as KIL-2 (Kemijski Institute Ljubljana) have received some attention [16,17]. Produced in Ljubljana at 2010, KIL-2 is a relatively novel mesostructured wormhole silicate support with interparticle and textural porosity [18]. Due to the novelty of KIL-2, a limited number of studies have explored its potential applications, and to the best of our knowledge, none reported on its effect on biomass pyrolysis and aromatics production. Popova et.al. used KIL-2 impregnated with different quantities of ZrO_2_ and sulphates to vary the acidity (both strength and type) of the catalysts for the catalytic esterification of glycerol with acetic acid [19]. Compared to pure zirconia, KIL-2-supported zirconia proved to be more active and efficient for the esterification reaction. In other research, Popova et al. compared Co/KIL-2 with Co/SBA-15 during the oxidation of toluene. It was concluded that the interparticle mesoporosity of KIL-2 made it an effective catalyst for the removal of toluene in the gas phase. It was also concluded that compared to the SBA-15, the lower cost, stability, and high catalytic activity of KIL-2 made it a catalyst with higher potential to remove the volatile organic compounds [20]. Therefore, there is interest in evaluating KIL-2 as a catalyst for biomass pyrolysis; so to study its effect on the pyrolysis of biomass in a more isolated manner, it was decided to study four feedstocks (cellulose, phenylalanine, lignin and wool waste) representing cellulosic and protein-rich materials in the first two and industrial lignin and nitrogen-rich waste in the latter two. Phenylalanine was selected because of its broad functional groups, which represent not only the carboxylic acid group, but also aromatics in protein-rich feedstocks. Some studies focused on phenylalanine uncatalyzed pyrolysis from 450 to 950 °C as being a key component of tobacco [21,22]. Two main decomposition pathways were identified: (i) decarboxylation whereby separation of the carboxyl group, nitrile amides are formed and (ii) cinnamic acid forms by the removal of the amine group (deamination). Paterson isolated the products obtained from both paths and concluded that the decarboxylation path is the dominant one with the deamination path having a limited degree of effect [21]. Cellulose is a polysaccharide of glucopyranose units. Based on the data in the literature, the main decomposition of cellulose starts from 300 °C and if the temperature is increased to 370 °C, a weight loss of 70% is the result of intermediate anhydro-cellulose formation [23]. As the temperature goes above 400 °C, the higher the chance of aromatic formation [24]. Additionally, water will be lost at low temperatures, which can affect the final products by facilitating benzene formation by providing the ideal conditions for the generation of carbon-to-carbon unsaturated bonds [25]. As the temperature increases to around 400 °C, the depolymerisation is further promoted and compounds such as levoglucosan and levoglucosenone, generally anhydro-saccharides, are formed [26]. Like phenylalanine, these products will undergo secondary decomposition to generate secondary products. Lignin is a complex polymer made up of three phenyl–propane cross chains, coniferyl alcohol, sinapyl alcohol, and coumaryl alcohol. Due to the complexity of the molecule, the initial decomposition temperature can have a wide range, typically between 200 and 450 °C. The initially decomposed compounds are expected to be mostly incondensable low molecular weight compounds resulting from the breaking of the weak propyl chains between the monomers [27]. The mentioned properties such as low cost, high surface area, efficiency in removal of volatiles and the capability of being functionalised by metals, makes KIL-2 a promising support for catalysed biomass conversion and further research is needed to explore their potential applications in biofuel production. Therefore, two different KIL-2 based catalysts, one loaded with aluminium (Al-KIL-2) and one loaded with lithium (Li-KIL-2) were synthesised, characterised and their performance evaluate in comparison to a more established ZSM-5 catalyst in the pyrolysis of cellulose, lignin, phenylalanine and wool textile waste. Li and Al were selected as basic and acid modifying agents to evaluate their effect on the pyrolysis products’ yield and distribution.

## 2. Results

### 2.1. Catalyst Characterisation

The analyses carried out to characterise the catalysts are presented in Table 1. The BET surface area of Al-KIL-2 was by far the largest at 896 m^2^/g and in line with previous work [28]. Li-KIL-2 had by far the smallest BET surface area. It was highly probable that the disordered pores of the KIL-2 support were filled with lithium. This is because silica supports generally have pores which are larger than lithium. Furthermore, structural modifications could have occurred during the synthesis calcination stage. ZSM-5 catalysts showed an increase in the surface available from 362 m^2^/g (20-ZSM-5) to 376 m^2^/g (60-ZSM-5), a trend consistent to that observed by Shirazi et al. for increasing the Si/Al molar ratio [14]. Although the BET of the 30-ZSM-5 was almost identical to that of 20-ZSM-5, the experimental error in the readings for 20-ZSM-5 and 30-ZSM-5 were ±3.35 m^2^/g and ±4.65 m^2^/g, respectively, meaning the trend could still be observed. The decrease in surface could be related to the incorporation of Al in the framework of ZSM-5.

**Figure 1:**
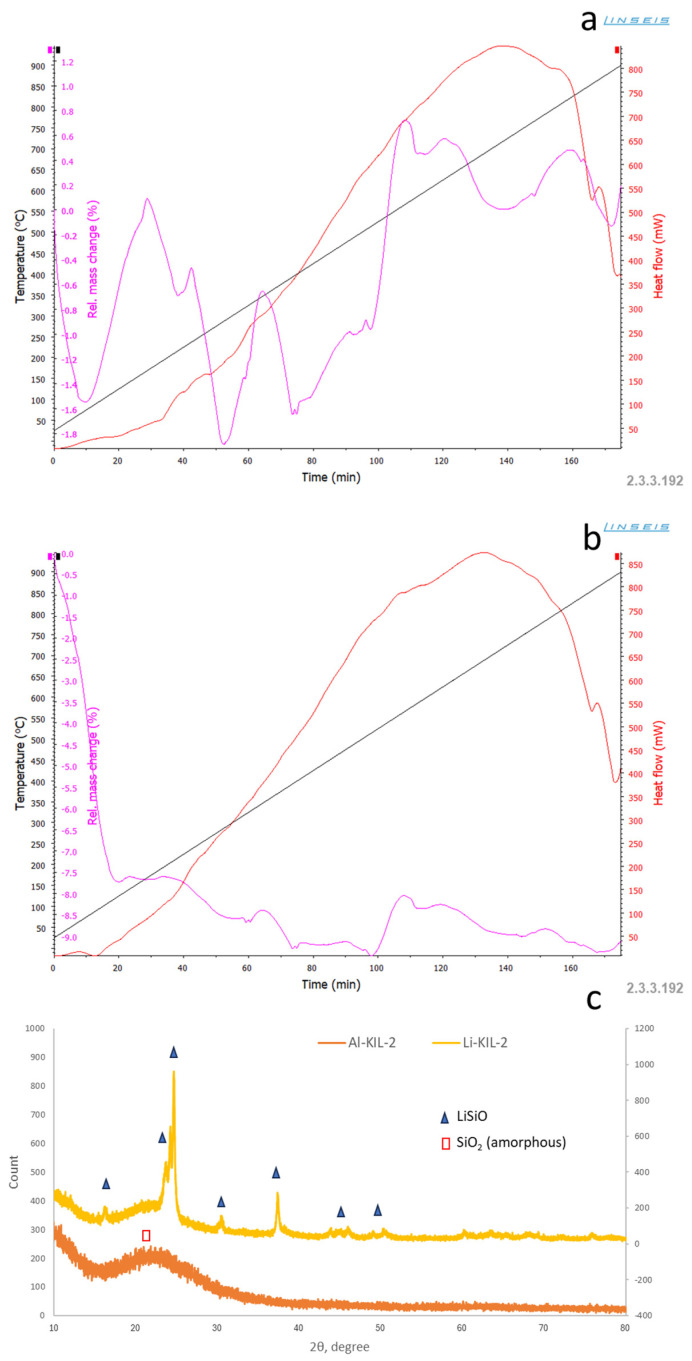
(**a**) Dynamic CO_2_ adsorption profile for Li-KIL-2; (**b**) dynamic CO_2_ adsorption profile for Al-KIL-2 and (**c**) XRD of KIL-2 catalysts.

The pore opening is a parameter that can greatly affect the catalytic activity allowing or obstructing access to the acid sites to the reacting species. ZSM-5 is well known to possess micropores, so that their median pore width was evaluated by the semi-empirical Horvath–Kawazoe processing method and resulted in micropores of about 0.55 nm. Macro-/mesopores for Li-KIL-2 and Al-KIL-2 were instead estimated by the Barrett, Joyner, and Halenda (BJH) procedure that calculates pore size distributions from experimental isotherms using the Kelvin model of pore filling. Hierarchic Al-KIL-2 resulted mostly in meso-/macropores (1.96 cm^3^/g between 1.7 and 300 nm) vs. micropores (0.19 cm^3^/g < 1 nm estimated by HJ) and a BJH median pore width of 7.9 nm. The HJ mesopore and micropore distribution for Al-KIL-2 can be appreciated in Figure 1c, where mesopore area (total of 842.5 m^2^/g) is larger for pore width between 15 and 6 nm and micropores account for 46 m^2^/g. The Li-KIL-2 also had mesopores with median pore width of 3.7 nm, but less significant surface. Therefore, Al-KIL-2 textural mesoporosity and microporosity could provide the prospect for enhanced diffusion of the reactants to the active sites and the products out of the larger pores, due to its high surface area and larger pores than ZSM-5.

The acidity of Al-KIL-2 and Li-KIL-2 was also evaluated by NH_3_-TPD. Al-KIL-2 had an acidity in between 20- and 30-ZSM-5, while Li-KIL-2’s acidity was very low. Li et al. (2019) found out that Li-KIL-2 showed both Brønsted and Lewis (in majority) acid sites originating from the framework Al and the epitaxially grown hyperbranched aluminosilicate, respectively [30]. The basicity and basic site strength of the Li-KIL-2 and Al-KIL-2 were evaluated by CO_2_ temperature-programmed sorption and the results are reported in Table 1 and Figure 1a,b, where the Li-KIL-2 had a much larger amount of mild and strong basic sites vs. the Al-KIL-2. XRD analyses were carried out to verify if the Al-KIL-2 and Li-KIL-2 were in amorphous phase as expected. Figure 1c shows the diffraction patterns of the two KIL-2 catalysts. As expected, the Al-KIL-2 catalyst denotes the absence of crystallinity, and it can be considered fully amorphous. In contrast, while Li-KIL-2 contains an amorphous SiO_2_ phase (00-029-0085), it also exhibits crystalline lithium silicon oxide (00-049-0803). This supports the idea that structural changes occurred in the KIL-2 during the calcination process.

The differences in morphology, mineral phases, acidity, basicity and porosity are expected to result in different behaviour during the biomass pyrolysis.

### 2.2. Model Compound Pyrolysis

The three model compounds were pyrolysed using both a thermo-gravimetric analyser (TGA) and a fixed bed reactor. TGA tests were carried out to evaluate the decomposition rates of the model compounds.

#### 2.2.1. Pyrolysis Using TGA

**Figure 2:**
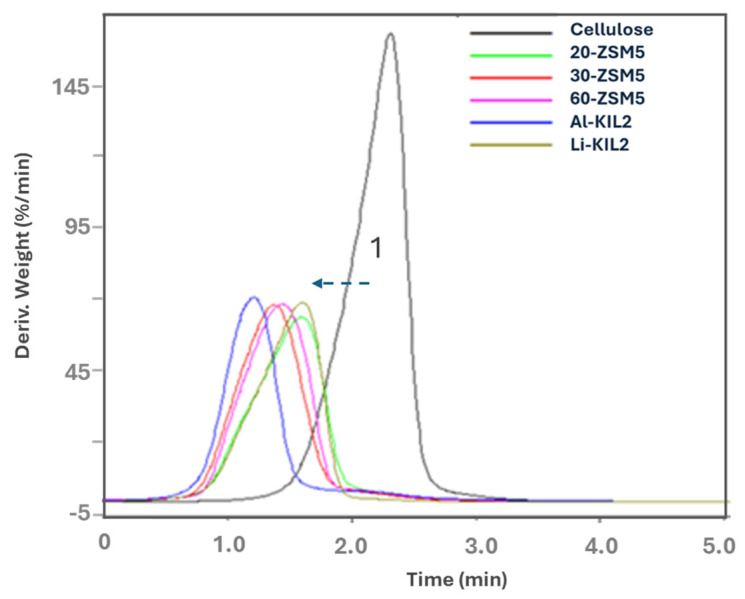
Cellulose DTG comparison curve.

Figure 2 shows the effect of the catalysts on derivative weight loss versus time for cellulose pyrolysis. Furthermore, Table 2 summarises the peaks, the starting temperature of pyrolysis, and the percentage loss for cellulose.

All the catalysts reduced the temperature at which the decomposition started (peak 1), meaning that the catalysts facilitated an alternative route with lower activation energy. Al-KIL-2 was the most effective in decreasing the decomposition temperature of about 30 °C and resulting in the fastest decomposition rate (71%/min) among the catalysts.

**Figure 3:**
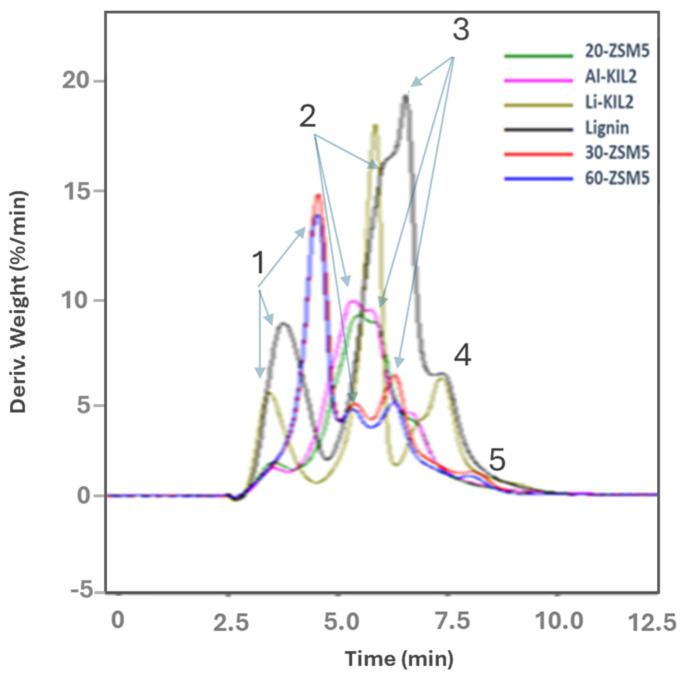
DTG of lignin with and without catalysts.

Figure 3 and Table 3 show the catalysts effect on the derivative weight loss versus time for lignin pyrolysis. The decomposition curves were more complex due to the nature of the lignin waste that also contain adsorbed water, hemicellulose and cellulose residues [27]. Peak 1 in Figure 3 is associated with the evolution of water that takes place between 80 and 115 °C for lignin waste and the KIL-2 catalysts, while it occurs at a higher temperature (150 °C) for the 30 and 60-ZSM-5. Hemicellulose and cellulose decomposition are represented, respectively, by peaks 2 and 3, while the remnant peaks 4 and 5 can be associated with lignin. The decomposition of lignin is a slower thermal event than those of cellulose and hemicellulose and typically occurs in a wide temperature range (280–600 °C) [27]. Among the tested catalysts, the basic Li-KIL-2 promotes a higher decomposition rate for lignin waste. In contrast, the more acidic Al-KIL-2 and 20-ZSM-5 were more effective than the less acidic 30- and 60-ZSM-5 catalysts. The acidity of the catalysts seems to have a direct relation to the decomposition temperature. For instance, the most acidic 20-ZSM-5 and Al-KIL-2 reduced the temperature of peaks 2 and 3.

**Figure 4:**
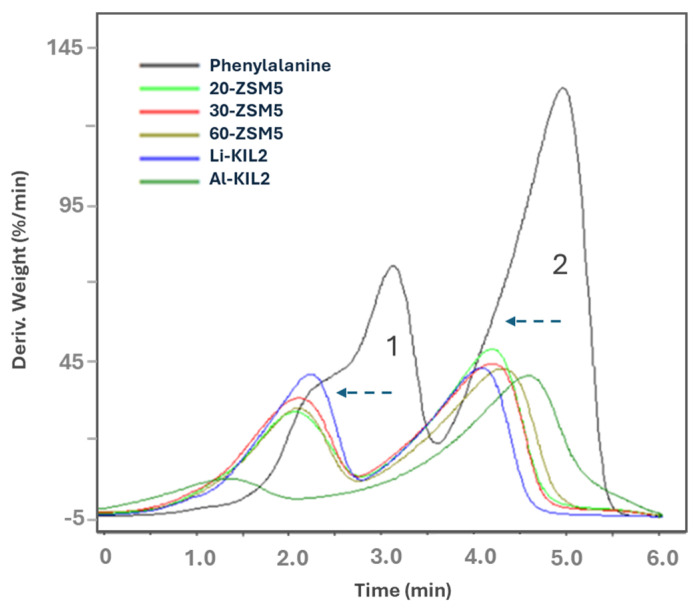
Phenylalanine DTG comparison curve.

Figure 4 and Table 4 summarise the effect of catalysts on derivative weight loss versus time for phenylalanine pyrolysis. All the catalysts reduced the decomposition initiation temperature by at least 100 *°C*, indicating a lower activation energy requirement. The peak 1 was likely to represent volatilisation of light, incondensable compounds such as CO, CO_2_ and NH_3_ and adsorbed water. The decomposition rate of this peak was increased by Li-KIL-2. The peak 2 represents heavier aromatics such as toluene and benzene-ethanamine [31]. The more acidic 20-ZSM-5 slightly favoured the decomposition of the second peak. Al-KIL-2 reduced the temperature at which the first peak occurred by about 40 °C and resulted in the lowest rate, while increasing the temperature at which the second decomposition occurred.

#### 2.2.2. Fixed Bed Pyrolysis Tests

**Figure 5:**
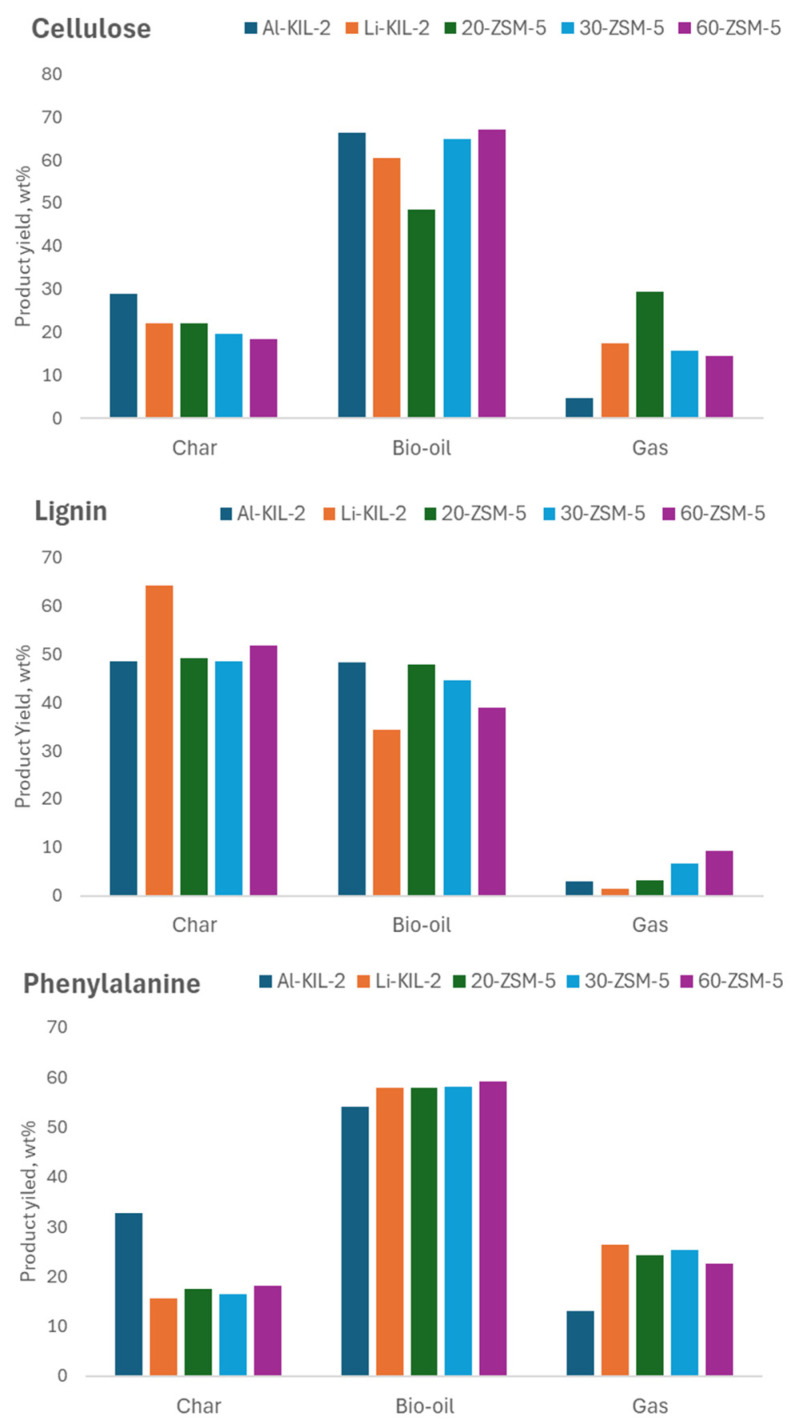
Mass balance from fixed bed experiments carried out at 500 °C.

Figure 5 reports the distribution of the products obtained in the fixed bed experiments in the presence of the three model compounds. In the case of cellulose, the use of catalysts increased the char yield compared to the non-catalytic runs, which can be linked to the catalysts’ acidity. In more detail, the amount of char produced in the fixed bed during uncatalysed pyrolysis of cellulose was less than 9% at 500 °C, which matches the values reported in the literature [32]. Among the tested catalysts, the production of char was the lowest when 60- and 30-ZSM-5 were used, compared to 20-ZSM-5, confirming that higher acidity promotes charring. Although Al-KIL-2 had an acidity just lower than 20-ZSM-5, it increased the char yield to 29 wt%. The difference in char yield between 20-ZSM-5 and Al-KIL-2 suggests that pore size and available surface also play a pivotal role in increasing coking reactions. This phenomenon is due to the condensation of higher molecular weight components produced during secondary reactions in the mesopores of Al-KIL-2 coupled to its acidity. The oil yield was reduced in the presence of the most acidic 20-ZSM-5 in favour of gas products such as CO_2_ via decarboxylation. In support of this observation, it was shown that zeolite with lower SiO_2_/Al_2_O_3_ mole ratio are more effective on decarboxylation [33].

As expected, char production was larger for lignin pyrolysis compared to cellulose due to its more complex structure of cross-linked polymers. The zeolite acidity significantly enhanced the conversion of lignin to char compared to pyrolysis without a catalyst, which yielded 28 wt% char [27]. However, no noticeable differences in char yield were observed with changes in the Si molar ratio. On the other hand, the basic Li-KIL-2 performed poorly in the pyrolysis of lignin, resulting in a very high char yield. Dalluge et al. observed increased char yields when adding alkali metals (Li, Na, K and Cs) to lignin instead of alkali earth metals (Ca, Mg), indicating that increased electro-positivity increases the catalytic activity of metal cations toward char production [34].

Overall, phenylalanine pyrolysis produced a higher bio-oil yield than lignin but lower than that from cellulose. The gas yield was generally greater among the three feedstocks; however, it decreased in the presence of Al-KIL-2, leading to an increase in char production.

**Figure 6:**
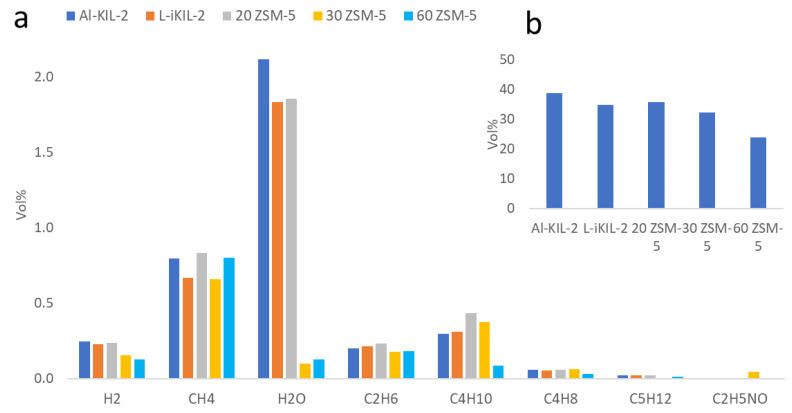
(**a**) Gas analysis of cellulose catalytic pyrolysis in fixed bed; (**b**) CO_2_ content in gas.

As shown in Figure 6b, all the catalysts promoted decarboxylation, with Al-KIL-2 producing the highest CO_2_ yield. The amounts of other gas components were quite small compared to CO_2_. Carbon monoxide (CO) was not quantified because its signal overlaps with that of nitrogen, which was used as the carrier gas. The acidity of the catalysts appeared to influence hydrocarbon yields, with 20-ZSM-5 generating more H_2_, CH_4_, C_2_H_6_, and C_4_H_10_. Additionally, the catalysts facilitated deoxygenation in the form of water [35].

**Figure 7:**
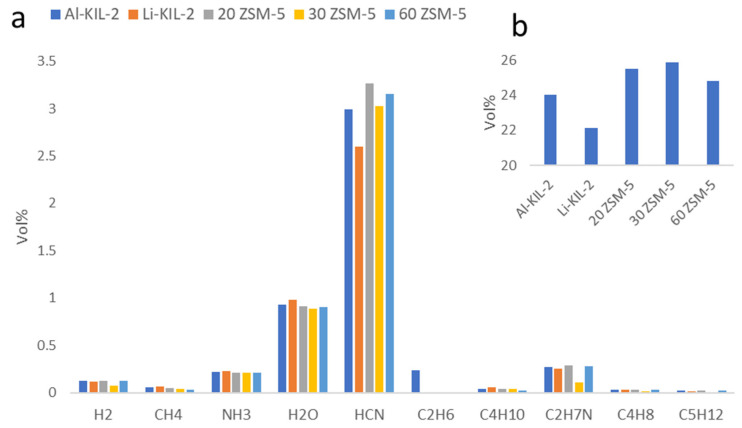
(**a**) Gas analysis of phenylalanine catalytic pyrolysis in fixed bed; (**b**) CO_2_ content in gas.

CO_2_ was the primary gas product from the catalytic pyrolysis of phenylalanine (Figure 7b), with ZSM-5 producing the highest amounts. Deamination of phenylalanine typically yields cinnamic acid [36]. One possible pathway for CO_2_ generation is the decomposition of cinnamic acid and other intermediate compounds. Forman et al. reported a CO to CO_2_ ratio of 2 to 1 through this pathway [31], but we were unable to measure CO production in our experiment to confirm this.

The product gas contained nitrogen-rich compounds such as ammonia, HCN, and C_2_H_7_N, but the relationship between catalyst acidity and nitrogen compounds was unclear. However, the basicity of the catalyst appears to correlate with HCN levels in the gas. Specifically, the percentage of HCN decreased by about 15% in the presence of Li-KIL-2 compared to Al-KIL-2, which is consistent with Li-KIL-2’s stronger basicity. This trend aligns with previous research, which found that a composite catalyst with an Fe ratio of 1:3 suppressed HCN by 53.6% during the catalytic pyrolysis of proline [13]. 

The GC-MS results of the bio-oils were categorised by functional groups to gain a better understanding of the catalysts’ activity and link it to their properties, with analyses conducted for each model compound.

As shown in Table 5, the ZSM-5 catalysts performed as expected from the literature, effectively converting cellulose and promoting aromatic generation. The quantity of aromatics increased with acidity, following this order: 20-ZSM-5 > 60-ZSM-5 > 30-ZSM-5. The large mesopore surface area of Al-KIL-2 made it the most efficient catalyst for aromatic production, indicating that accessibility to acid sites for the cracked volatiles is a crucial factor. Cyclo-propane-methanol, 1-phenyl- (C_10_H_12_O), 4-Methyl-4-phenyl-2,3:5,6-diepoxycyclohexan (C_12_H_16_O), bis(1-phenylethyl)ether (C_16_H_18_O) and 1,4-Diphenyl-1,3-butadiene (C_16_H_14_) were the most abundant aromatics, respectively, with 9, 6, 5 and 4 area%, suggesting Al-KIL-2 is efficient in deoxygenating the pyrolysis volatiles from cellulose, but also promotes coke formation as shown in Figure 3.

As an opposing trend, the Li-KIL-2 catalyst was active only in the depolymerisation of cellulose to sugars, while clearly hindering the dehydration and aromatisation processes, but instead promoted ketonisation and alcohol formation, typical for basic catalysts.

Considering the molecular structure of cellulose and the existing literature on its pyrolysis pathways, the initial stage involves dehydration, leading to the formation of anhydro sugars such as anhydro-oligosaccharides (classified as carbohydrates in this paper) [31]. In the presence of acid catalysts, these dehydrated carbohydrates can undergo further dehydration, decarboxylation, and reforming (oligomerisation) to produce furans (classified as ketones in this paper), aldehydes, aromatics, and low molecular weight olefins (C_3_–C_6_) [21].

The significant presence of carbohydrates in the oils from Li-KIL-2, 30-ZSM-5, and 60-ZSM-5 indicates that they performed well in the initial stages of the process to produce dehydrated carbohydrates. However, they exhibited limited secondary reactions and aromatisation capability due to their lower acidity. This is further supported by the quantity of alkenes produced. One competing pathway for aromatic production is the formation of olefins (alkenes) [35]. As shown in Table 5, Al-KIL-2 produced very few alkenes compared to aromatics. The larger amount of polycyclic aromatic hydrocarbons (PAHs) confirms Al-KIL-2’s greater tendency toward coking reactions.

The yield of esters from Al-KIL-2 was nearly double that of the other catalysts, indicating that its mild acidity and large accessible mesopore surface facilitated the esterification of carboxylic acids derived from cellulose. Popova et al. (2017) demonstrated that acidic, sulphated Zr-KIL-2-type silica was effective in the esterification of levulinic acid in the presence of alcohol, attributing this activity to the good dispersion of Zr within the KIL-2 mesopores [37]. As expected, due to the molecular structure of lignin, aromatics were a larger constituent of the products compared to cellulose, as can be seen in Table 6. During the non-catalytic pyrolysis of lignin, the first step is the thermal depolymerisation that produces alkoxy phenols or small oligomers [38]. This is followed by the conversion of these large alkoxy phenols and oligomers to smaller ones through cracking, dealkylation and dehydration. These smaller components can either be converted to alkyl phenols through dealkoxylation, isomerisation and extra oligomerisation or small olefins (C_2_ to C_6_) through cracking and dehydration [31]. The products from either path can further react together to form other mono- or polyaromatics through oligomerisation. Examining the trends in overall conversion to aromatics, there was a clear direct correlation between acidity and aromatics production. Li-KIL-2 significantly favoured the production of phenols compared to the other catalysts. As mentioned earlier, the initial step in lignin pyrolysis is depolymerisation, leading to the formation of large alkoxy phenols. In contrast, the ZSM-5 catalysts and Al-KIL-2 were able to dehydrate and crack lignin, followed by aromatisation. Cis-stilbene (C_14_H_12_), 1,2-diphenylcyclopropane (C_15_H_14_), 2-phenethyl-β-phenylpropionate (C_17_H_18_O) and benzeneethanol, β-methyl- (C_9_H_12_O) were the most abundant among the aromatic compounds, with the phenyl group present in many of the identified compounds. The acidic 20- and 30-ZSM-5 also resulted in similar compounds (benzene, 4-pentenyl-; 2-phenethyl-.beta.-phenylpropionate; 2-propanone, 1,3-diphenyl-; benzene, (1,2,2-trimethyl-3-butenyl)-and bibenzyl).

When comparing the products of the acidic Al-KIL-2 and Li-KIL-2, a key observation could be made. While the latter resulted in 9 area% alkyl phenols (2-methylphenol, 4-ethylphenol), Al-KIL-2 and the acid ZSM-5 zeolites were rich in cis-stilbene, an aromatic alkene, which was likely to have been produced by dehydration and further oligomerisation of two 2-methylphenol-like intermediates. This shows that the acid sites of the catalysts were determinant to deoxygenate the phenolics and in condensation reactions. Interestingly, the acidity of ZSM-5 catalysts had direct proportionality in the production of stilbene. In more detail, all the ZSM-5 catalysts had stilbene in their top three products with the highest amount obtained in the presence of 20-ZSM-5, which is consistent with the acidity strength. This is also confirmed by the abundance of polyaromatics, which was highest for the 20-ZSM-5 of the three zeolites. The largest content for Al-KIL-2 can be ascribed to its larger porosity and pore size. The Al-KIL-2 also resulted in being very efficient at reducing phenols and comparable to the ZSM-5 and recently developed Nb-modified ZSM-5 [11].

During the pyrolysis of phenylalanine, the following mechanism can typically be expected: decarboxylation, homolyses, cyclisation, deamination. An initial benzylic bond break may be followed by the formation of intermediates such as phenyl and unstable molecules [31]. During homolyses, CO_2_ is released producing unstable compounds, which may be followed by the removal of H_2_. These two steps may lead to the generation of aromatic nitriles. The deamination path followed by homolyses can lead to the production of benzene derivatives such as styrene (C_8_H_8_). If hydrogen radicals are available, other aromatic compounds may be formed through addition, reformation, and cyclisation. The addition of carboxyl to phenylalanine may result in the production of aromatic aldehydes. On the other hand, the removal of the carboxyl group will produce a compound with an amine radical, which may produce indoles through reformation [31]. From the phenylalanine monomer and previous works, cyclic and aromatic compounds were expected to be dominant from the phenylalanine pyrolysis [39]. Table 7 illustrates the product component distribution results for the GC-MS of the oil. The dominance of aromatics in the oil products fell in line with expectations. Comparing the ZSM-5 catalysts, there did not seem to be a significant difference between the overall percentage of aromatics of 60-ZSM-5 and 30-ZSM-5.

However, despite the acidity of the three catalysts, there was an inverse correlation with overall aromatic production. Additionally, when considering nitrogen-free aromatics, a direct correlation was observed between the Si/Al ratio and aromatic yield, with 60-ZSM-5 being the most effective at removing nitrogen from the aromatics. Al-KIL-2 excelled in both nitrogen removal and producing the highest yield of aromatics. GC-MS analyses revealed a wide variety of components in the oil product, indicating that the reactions were not selective for just a few major compounds. For example, the oil product from phenylalanine pyrolysis with Al-KIL-2 contained over 80 different components. Comparing the most abundant compounds for ZSM-5 catalysts, the acidity of the catalyst seemed to be important in deoxygenating phenylalanine. This was based on comparing the amount of oxygen in the top three products of 20-ZSM-5 with those of the other two ZSM-5 catalysts. Furthermore, acidity favoured the production of smaller numbers of components with a higher intensity. For instance, only three components made up over 25% of aromatics in the more acidic 20-ZSM-5, while this amount for the same quantity of components was 17% for 30-ZSM-5. Two phenomena were dominant in the presence of Al-KIL-2; the first one was the minimisation of small compounds such as alkenes, while maximisation of complex aromatics occurred. However, the Al-KIL-2 catalyst, due to the large surface area available, proved its capability to break down the phenylalanine monomer and form more complex components. One explanation for the production of components such as “[1.2.4] Triazolo[4,3-a] quinoxaline, 1,4-dimethy” could be the selectivity of Al-KIL-2 for decarboxylation followed by cyclisation and oxidation (removal of H). In the run with Li-KIL-2, the component with the highest quantity was 7-azabicyclo[4.2.2]deca-2,4,9-trien-8-one (C_9_H_9_NO). This could be due to the selectivity of Li-KIL-2 for a path in which the hydroxyl group was removed through a carbocation intermediate followed by a benzene ring cleavage. Another notable finding from the analysis of the most abundant compounds was the presence of oxygen in all of them. This suggests that Li-KIL-2 has low selectivity for the removal of hydroxyl groups and reduction. When comparing the two KIL-2 catalysts to ZSM-5, the KIL-2 catalysts not only promoted the production of aromatics over other components, such as alcohol (with Al-KIL-2), but also performed better in deoxygenating and denitrogenating the oil product. This indicates that the supported KIL-2 catalysts could be well-suited for the catalytic pyrolysis of protein-rich feedstocks.

Previous studies support this idea; for instance, Ti-HMS/KIL-2 demonstrated good adsorptive denitrogenation performance for quinoline and pyridine [40]. Quinoline, being a larger molecule, requires a larger surface area for effective adsorption. Consequently, the lower levels of nitrogen-containing aromatics obtained with Al-KIL-2 may be attributed to its larger surface area compared to Li-KIL-2, in addition to its meso-/microporosity. The adsorption of nitrogen-containing heterocycles could also explain the higher char yield observed for the KIL-2 catalysts (see Table 7), which would necessitate thermal regeneration at temperatures exceeding 500 °C.

### 2.3. Pyrolysis of Textile Wool Waste

**Figure 8:**
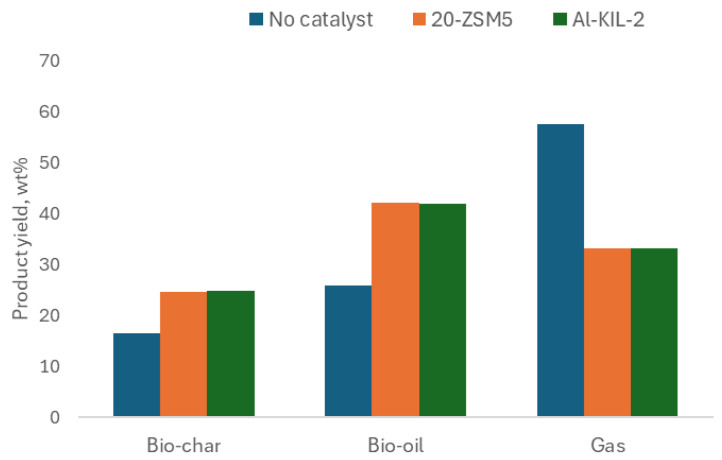
Product distribution comparison of catalytic and non-catalytic samples in fixed bed.

The effect of 20-ZSM-5 and Al-KIL-2 on modifying the pyrolysis products was evaluated by pyrolyzing wool textile waste at 500 °C. As illustrated in Figure 8, Al-KIL-2 and 20-ZSM-5 increased the fraction recovered in bio-oil but also increased the char yield. This may be linked to the large surface of Al-KIL-2 and the presence of macro-/mesopores and mild acidity vs. micropores and the strong acidity of 20-ZSM-5, which favoured the conversion of large biomolecules.

**Figure 9:**
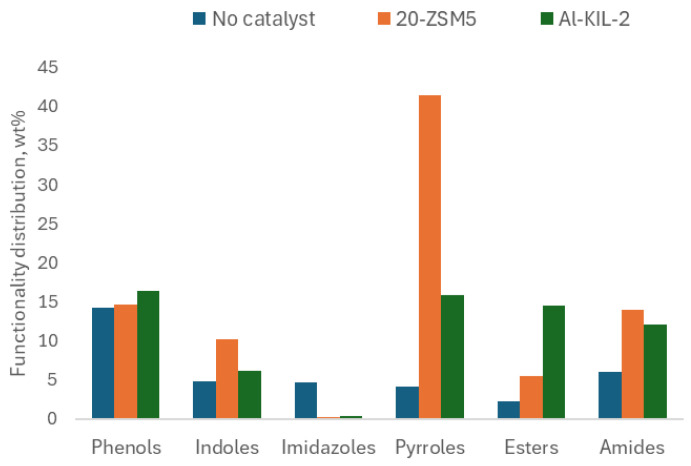
Effect of catalysts on oil’s most abundant functionalities (data from GC-MS).

The number of unique compounds in the bio-oil significantly decreased with the use of the 20-ZSM-5 and Al-KIL-2 catalysts, dropping from about 150 (without catalysts) to 90 and 50, respectively. Meanwhile, the proportion of the ten most abundant compounds increased from 35% to around 75% for both catalysts. As shown in Figure 9, the catalysts effectively enhanced the production of aromatics, particularly indoles, pyrroles, and amides. The increase in indoles suggests that the catalysts are capable of breaking down the carbon bonds in the indigoid chromophore.

One specific pyrrole, “Pyrrolo[1,2-a] pyrazine-1,4-dione, hexahydro-3-(2-methylpropyl)”, was present in all three samples and was the most abundant compound in the 20-ZSM-5 sample. This compound may be formed by removing hydroxyl groups from several proline amino acids within the wool structure. The catalysts did not show significant differences in amide production; however, esters were predominant in the Al-KIL-2 sample. Esters and carboxylic acids likely resulted from the cracking of various amino acids and dye molecules such as phenylalanine.

**Figure 10:**
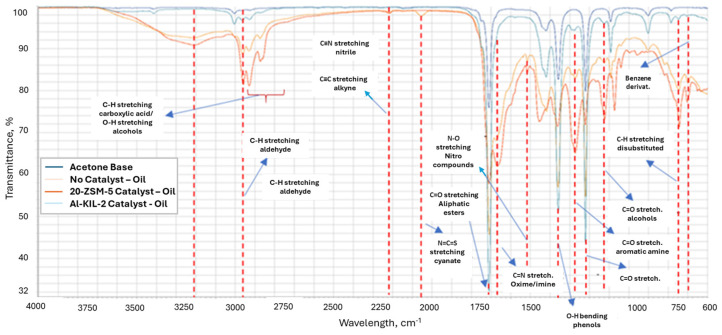
FTIR of catalytic and non-catalytic oil products.

FTIR analysis (Figure 10) provides a comprehensive overview of the functionalities present in the bio-oil. The transmittance band between 2050 and 2075 cm^−1^ indicates the presence of cyanates in the non-catalytic bio-oil, but this band disappears when catalysts are used during pyrolysis. The band between 1650 and 1700 cm^−1^ suggests the presence of an oxime, which is not observed in the Al-KIL-2 sample, aligning with the GC-MS analysis results.

The formation of imines typically occurs when primary amines react with ketones or aldehydes in the presence of an acidic catalyst. The more prominent peak in the 20-ZSM-5 sample compared to the non-catalytic run indicates that the acidity of the catalysts plays a significant role. Furthermore, the high quantities of esters produced with Al-KIL-2 suggest that this reaction pathway was not favoured by this catalyst. Additionally, the catalysts influenced the quantity of C-H groups at different wavelengths, reflecting their varying effects on the bio-oil composition.

These groups increased in the oil samples with the use of 20-ZSM-5 compared to the non-catalytic tests, while Al-KIL-2 seemed to reduce it. Another major peak occurring between 1350 and 1400 cm^−1^ belongs to the O-H group in phenols, which increased in the order Al-KIL-2 > 20-ZSM-5 > non-catalytic. Overall, the data suggested that the use of 20-ZSM-5 resulted in an increase in nitrogen-containing compounds in the oil. This was observed in GC-MS results where the “Pyrrolo[1,2-a] pyrazine-1,4-dione, hexahydro-3-2-methylpropyl” quantity was more than quintuple that of Al-KIL-2 and triple the non-catalytic run, while pyrroles and indoles were the dominant groups. Furthermore, FTIR peaks illustrated that imines/oximes and amines were present in the oil. On the other hand, GC-MS and FTIR data suggest that Al-KIL-2 promoted the generation of esters and compounds with oxygen-containing functional groups.

## 3. Materials and Methods

### 3.1. Materials and Material Synthesis

Microcrystalline cellulose powder (Prod. No: 310697) with particles size < 20 µm and phenylalanine (Prod. No: PHR1100) were acquired from Sigma Aldrich. Industrial waste lignin was procured from a bioethanol plant in Sweden (Etanolteknik AB, Örnsköldsvik, SE) and contained 51 wt% C, 5.7 wt% H, 1.6 wt% N and 37.7 wt% O [27]. Since it was obtained from ethanol production by a 2-stage weak acid hydrolysis of softwood, it was expected to have a degree of degradation compared to untreated lignin [41]. Wool textile waste was provided by Harris Tweed Authority (Stornoway, UK). Mesostructured silica KIL-2 catalysts were prepared as previously described with some slight modifications [17]. Synthesis reagents were acquired and utilised as received from Sigma-Aldrich/Merck. Tetraethyl orthosilicate and triethanolamine homogenous solution was mixed with an aqueous solution of tetraethylammonium hydroxide for gel formation. After drying, a solvothermal treatment was carried out using ethanol for 48 h at 150 °C, using Teflon-lined steel autoclaves. The obtained powder was then finally calcined at 800 °C for 10 h to obtain the final KIL-2 silica parent material. Li-KIL2- and Al-KIL-2 were prepared using lithium nitrate (LiNO_3_, 99.99%, Merck, Darmstadt, Germany) and aluminum chloride hexahydrate (AlCl_3_ · 6H_2_O, 99%, Aldrich), respectively. The materials were prepared by using a planetary ball mill (Pulverisette 6, Fritsch) during 10 min at 350 rpm. Then, the obtained powders were calcined at 700 °C for 5 h. The theoretical molar ratios were Li/Si = 10 and Al/Si 10. ZSM-5 was synthesised following a procedure previously reported [42]. The synthesised materials were then processed in autoclaves for 24 h at 180 °C. The solid products were filtered, washed with water, and dried in an oven at 60 °C for 24 h.

### 3.2. Catalyst Characterisation

#### 3.2.1. Surface Analysis

Surface area analysis was conducted using the Micromeritics Gemini VII 2390 Surface Area Analyser. BET surface area and Horvath–Kawazoe pore volume and median pore width, and, both Barrett–Joyner–Halenda (BJH) adsorption area and volume were obtained. Before the experimental run, each sample was degassed for four hours at 150 °C to remove any impurities and water, which could be present. The sample of catalyst was weighed before and after degassing using a scale accurate to four decimal points. All the surface area experiments were taken at 77 K, hence the use of liquid nitrogen. Nitrogen adsorption was measured along an isotherm between partial pressures of 0.05 and 0.35 as this is where the isotherm is maintained. Horvath–Kawazoe equation was used for determining the pore volume and median pore width for micropores. It measures the interaction between the nitrogen adsorbate and the pore walls. This is performed in the same range of partial pressures as BET surface areas. The model applies a correlation between pores of given width being filled at partial pressures [43]. The BJH adsorption method was used to determine the maximum and minimum values for both the pore volume and pore area and mesopore dimension.

#### 3.2.2. Acid Site Analysis

The acidity strength was analysed by temperature programmed desorption (TPD) at the Quantachrome’s materials characterisation laboratory using 10% pyridine analyte in a Chem Star Tpx chemisorption analyser with a calibration loop of 532 µL; 50 °C; 1 atm.

#### 3.2.3. Basicity Assessment

The basicity was calculated by running a dynamic CO_2_ adsorption test using a Linseis STA PT 1600 model (Linseis Messgeraete GmbH, Selb, Germany). The temperature programme involved raising the temperature from ambient to 900 °C in a 100% CO_2_ atmosphere (30 mL/min).

#### 3.2.4. XRD Analysis

The XRD analysis was carried out using a Bruker X8-Apex2 CCD diffractometer (Bruker, Billerica, MA, USA).

### 3.3. Pyrolysis Methodology

After analysing the catalysts on their own and determining their properties, their effectivity in pyrolysis was tested. To achieve this, two different types of experiments were carried out. Initially the catalysts were run in TGA-DTG (thermogravimetric-derivative thermogravimetric analysis) in small quantities and the amount of liquid and vapour was determined. Afterwards, to observe if the effect of the catalysts on the model compounds is deferred by increasing the size of the procedure, a fixed amount was used to run the same experiment at a larger scale. The results of these two methods were compared.

#### 3.3.1. TGA-DTG

The TGA-DTG analysis took place using a TA TGA Q500 with N_2_ flowing at 100 mL/min and the following temperature program: the sample was kept isothermal at ambient temperature for thirty minutes and then increased by 100 °C/min till it reached 500 °C. It was then kept isothermal for a further 15 min before cooling down to ambient temperature again. The samples for the TGA were kept very small, typically between 15 and 20 mg. In the runs that were undertaken with catalyst and sample, the ratio was kept 1:1 on a mass basis and then thoroughly mixed.

To conduct the experiment, first the sample trays were cleaned using a burner and then individually tared within the pyrolysis chamber. The samples were then weighed, and the trays filled. The gas flow was set to 20 mL/min of CO_2_ and the vapour outlet cable was attached to the MS analyser with filament switched on and heated.

To determine the water weight, the first run entailed using the solo catalyst; this was to determine the change in weight % of the catalyst over the pyrolysis temperature range

#### 3.3.2. Fixed Bed Reactor

While fast pyrolysis occurs between 400 °C and 650 °C, a temperature of 500 °C was used for these experiments, with a residence time of 3 s. The masses of char, oil and gas were measured. The oil yield was collected in four Drechsel flasks and weighed; three were submerged in an ice bath with sodium chloride (NaCl) (−15 °C) and a fourth in liquid nitrogen. The masses of the solid residues were also weighed, and the resulting yields of char and gas determined by difference through mass balance as in the following equation:

Gas = 100 − (Bio-oil + Char).(1)

**Figure 11:**
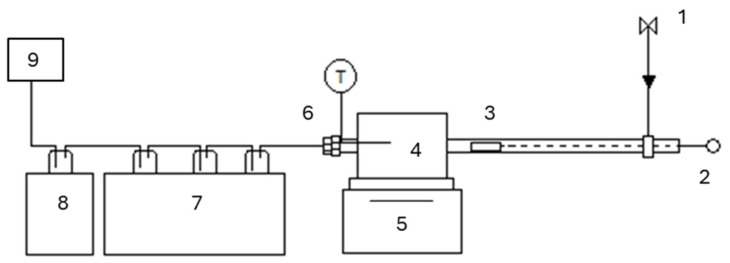
Scheme of in situ fixed bed used for the pyrolysis experiments.

Figure 11 shows the fixed bed reactor layout, which comprised a nitrogen gas inlet (1), a sample insertion system (2), a sample holder (3), a furnace (4), a tubular reactor with a heated section into the furnace (5—reaction chamber) and a bio-oil condensation system (7, 8) and a gas analyser (9). The temperature into the reaction chamber was controlled by a K-type thermocouple.

**Figure 12:**
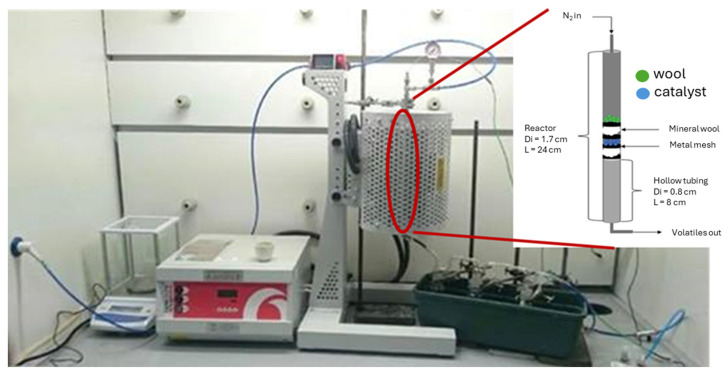
Ex situ fixed bed configuration used for the textile wool waste pyrolysis.

The sample and catalyst were measured individually on a scale accurate to four decimal points. The catalyst to sample mass ratio would be 50:50. A portion of 0.35 g of each was taken and then mixed and ground together. The resulting mixture was added to the sample boat and the total mass of the boat measured. The sample boat was loaded upstream of the reactor (7, in Figure 11); the purpose of keeping the boat upstream was to keep the boat out of the reactor until the appropriate temperature had been reached. This would allow for the sample to achieve fast heating rate (~1000 °C/min). The unit was sealed and a purge of N_2_ was run for ten minutes at a rate of 40 cm^3^/min. The flowrate of N_2_ was kept at this constant value throughout the experiment to maintain an oxygen-free environment. When the thermometer measuring the reaction chamber temperature reached 500 °C, the sample boat was inserted into the chamber and then the heating temperature was lowered to 550 °C. The sample was kept in the chamber for twenty minutes to allow complete reaction. After allowing sufficient time to cool the system (≈2 h), the reactor was opened, and the sample boat was removed and weighed. The four Dreschel bottles were detached from each other and weighed. The char was collected in a sample bag and the Dreschel bottles were washed with acetone to collect any oils. The oil/acetone mixture was then collected in small sample vials. The oils were then sent off to undergo GC-MS analysis. The fixed bed reactor was modified to run ex situ experiments in the presence of textile wool waste (Figure 12). The configuration consisted of a vertical furnace, a tube reactor where wool (0.5 g) and catalyst (0.5 g) were packed and kept separated by some loose mineral wool. Compared to the in situ case, the flowrate of injected nitrogen was increased (doubled to 100 mL/min). Pyrolysis temperature was set at 500 °C with a heating rate of 100 °C/min. Reactor was kept at this temperature for 20 min to ensure complete volatilisation. All the pyrolysis experiments were run in duplicate and averages were considered in the figures/tables. The error was found to be in the 1–6% range.

### 3.4. Bio-Oil Characterisation

The elemental analysis of the bio-oils was carried out using a Exeter CE-440 Elemental analyser (Exeter Analytica, North Chelmsford, MA, USA) in order to define the amount of carbon, hydrogen, nitrogen and oxygen (by difference). The GC-MS (gas chromatography–mass spectrometry) analysis of the bio-oils was carried out using a GC 8000 series equipped with VG Trio 1000 (Hewlett Packard, Palo Alto, CA, USA) in duplicate. The column (length: 30 m, inner diameter: 0.250; film: 0.25 µm) had temperature limits between 40 °C and 300 °C. The results were received in the form of pdf format with the chemical composition of each oil and its individual weighting in the sample. The oils were then classified by individual functional groups (alcohol, aldehyde, ketone, carboxylic acid, ester, ether, furan, mono-aromatic, polycyclic aromatic, nitrile, amine, nitrogen aromatic and nitrogenates).

The GC-MS data that were provided also gave an average of 200–300 individual components for each bio-oil sample. The compounds identified allowed an insight into the individual molecular trends of each catalyst. For each catalyst and model compound, the main compounds with a minimum of 2% area were considered. It should be noted that the GC-MS graphs received contained some impurities such as phthalates leached out from the GC capillary column, which were removed from the raw data set prior to categorising the oil sample components into functional groups for a clearer comparison between the catalysts. For each model compound, the raw GC-MS graph for each catalysts’ oil product were staggered on top of each other with vertical red lines across them which indicated the same time on all the graphs to simplify their comparison. FTIR analyses were carried out using a PerkinElmer (Shelton, CT, USA) Frontier and the transmission band identification was carried out based on Ref [44].

## 4. Conclusions

In this work, Al- and Li-doped mesoporous KIL-2 were evaluated for the in situ catalytic pyrolysis of cellulose, lignin, phenylalanine and textile wool waste and compared to ZSM-5. Amorphous Al-KIL-2 had a large BET surface area (896 m^2^/g) with mesopores of BJH median pore width of 7.8 nm and acid sites of weak/mild strength, while Li-KIL-2 resulted in both amorphous and LiSiO crystalline phases and strong basic sites. Al-KIL-2 increased the char yield by 10 wt% compared to the other catalysts mostly due to its large surface and was by far the superior option for the aromatisation of the oil product. In more detail, the aromatic content of the Al-KIL-2 oil sample was over 12 times of that of Li-KIL-2 in the case of cellulose, and comparable to 20-ZSM-5 for lignin and the protein-rich materials. Furthermore, it minimised the oxygen-containing groups such as alcohols and ketones. Li-KIL-2 instead lacked aromatisation activity due to low acidity and available surface. Al-KIL-2 also increased the wool waste bio-oil yield, compared to the yield in the absence of catalyst, being also the most effective in reducing the number of unique compounds while increasing the concentration of pyrroles, esters and amides. Both Al-KIL-2 and ZSM-5 bio-oils from lignin pyrolysis were rich in cis-stilbene and 1,2-diphenylcyclopropane (C_15_H_14_), aromatics that were likely to have been produced by dehydration and further oligomerisation of two 2-methylphenol-like intermediates. This showed that the acid sites of the catalysts were determinant to deoxygenate phenolics. Future work should focus on assessing the recyclability of the Al-KIL-2 catalyst and evaluating the feasibility of scaling up this process. Currently, the synthesis cost of the catalyst poses a challenge at this stage of development.

## Figures and Tables

**Table 1:** Summary of the catalysts’ properties.

Properties	Unit	Li-KIL-2	Al-KIL-2	20-ZSM-5	30-ZSM-5	60-ZSM-5
BET (surf. area)	m^2^/g	23.9	896	362.0	361.8	376.9
Max. pore volume (H-K)	cm^3^/g	0.006	0.369	0.164	0.167	0.173
Median pore width (H-K)	nm	3.68 *	7.86 *	0.55	0.55	0.55
Total acidity	mmol/g	0.04	0.46	0.53 **	0.34 **	0.18 *
Mild basic sites (250–400 °C)	mmol/g	0.277	0.042	/	/	/
Strong basic sites (>400 °C)	mmol/g	0.536	0.223	/	/	/

* from BJH; ** from [29].

**Table 2:** Peak degradation for cellulose alone and in presence of different catalysts.

Cellulose		Peak 1
Base	Curve peak temperature °C	403
	Derivative weight (%/min)	80
Al-KIL-2	Curve peak temperature °C	377
	Derivative weight (%/min)	71
Li-KIL-2	Curve peak temperature °C	392
	Derivative weight (%/min)	70
20-ZSM-5	Curve peak temperature °C	392
	Derivative weight (%/min)	64.6
30-ZSM-5	Curve peak temperature °C	386
	Derivative weight (%/min)	68.7
60-ZSM-5	Curve peak temperature °C	389
	Derivative weight (%/min)	69

**Table 3:** Peak degradation for lignin alone and in presence of different catalysts.

Lignin		Peak 1	Peak 2	Peak 3	Peak 4	Peak 5
Base	Curve peak temperature °C	114.6	333.3	384.5	468.3	
	Derivative weight (%/min)	4.6	8.51	10.15	3.15	
Al-KIL-2	Curve peak temperature °C	86.6	334.6	377.1	468.8	
	Derivative weight (%/min)	2.1	10	9.5	4.2	
Li-KIL-2	Curve peak temperature °C	83.2	316.3	409.5	461.9	503
	Derivative weight (%/min)	5.5	19	3.8	6	0.5
20-ZSM-5	Curve peak temperature °C	84.0	339	377.3	465.5	
	Derivative weight (%/min)	1.8	9.3	8.9	3.8	
30-ZSM-5	Curve peak temperature °C	84.2	150	386.1	471.6	503.1
	Derivative weight (%/min)	1.7	15.4	4.7	6.1	1.2
60-ZSM-5	Curve peak temperature °C	84.3	150	383.9	478.4	503
	Derivative weight (%/min)	1.9	14.4	4.4	3.8	0.9

**Table 4:** Peak degradation for phenylalanine alone and in presence of different catalysts.

Phenylalanine		Peak 1	Peak 2
Base	Curve peak temperature °C	342.0	447.0
	Derivative weight (%/min)	23.5	45
Al-KIL-2	Curve peak temperature °C	266.8	428.0
	Derivative weight (%/min)	11.8	43.9
Li-KIL-2	Curve peak temperature °C	311.7	404.9
	Derivative weight (%/min)	44.3	46.2
20-ZSM-5	Curve peak temperature °C	303.7	410.6
	Derivative weight (%/min)	32.7	52.3
30-ZSM-5	Curve peak temperature °C	305.4	410.6
	Derivative weight (%/min)	37.0	47.6
60-ZSM-5	Curve peak temperature °C	304.9	415.2
	Derivative weight (%/min)	33.8	46.1

**Table 5:** GC-MS composition of bio-oil from cellulose catalytic pyrolysis.

			Area %		
Functional Group	Li-KIL-2	Al-KIL-2	20-ZSM-5	30-ZSM-5	60-ZSM-5
Alcohol	24.67	0.80	16.28	19.82	33.30
Aldehyde	2.46	0.26	2.05	0.50	0.21
Alkane	1.71	1.17	3.53	4.16	0.54
Alkene	3.79	4.12	8.12	4.27	0.05
Carbohydrate	30.61	2.78	19.36	39.05	40.97
Carboxylic acid	5.81	10.60	4.16	2.36	1.53
Ester	2.93	8.43	9.72	7.25	1.78
Ether	3.89	0.00	1.16	0.00	0.00
Furan	0.04	0.10	0.92	1.47	2.34
Ketone	20.87	3.55	14.58	15.10	9.03
Aromatic	3.21	63.36	18.49	6.02	7.63
Polycyclic aromatic	0.00	4.84	1.63	0.00	2.60
Error, %	3	5	3	5	6

**Table 6:** GC-MS composition of bio-oil from lignin catalytic pyrolysis.

			Area %		
Functional Group	Li-KIL-2	Al-KIL-2	20-ZSM-5	30-ZSM-5	60-ZSM-5
Alcohol	6.24	9.12	7.89	15.48	18.89
Aldehyde	0.90	0.00	0.93	0.00	0.12
Alkane	5.42	2.20	0.08	30.81	9.20
Alkene	1.04	8.50	12.37	5.40	6.90
Carboxylic acid	13.89	8.30	0.02	5.51	2.89
Ester	17.57	11.26	1.16	15.38	6.57
Ether	4.01	2.93	8.68	1.65	3.84
Ketone	7.15	2.46	3.14	3.77	2.85
Aromatic	25.00	36.20	49.30	15.80	36.70
Polycyclic aromatic	8.33	15.10	10.25	5.04	5.44
Phenols	10.00	3.80	5.10	1.00	4.40
Error, %	4	2	4	5	2

**Table 7:** GC-MS composition of bio-oil from phenylalanine catalytic pyrolysis.

			Area %		
Functional Group	Li-KIL-2	Al-KIL-2	20-ZSM-5	30-ZSM-5	60-ZSM-5
Alcohol	12.6	5.3	6.85	1.1	0.2
Alkene	9.6	0	1.2	5.4	3.0
Ketone	11.3	6.8	4.8	2.4	8.5
Nitrile	3.7	6.4	0.1	0.2	3.46
Nitrogen-free aromatics	50.0	63.0	28.0	30.1	45.0
Nitrogen aromatics	32.1	25.9	47.0	53.8	38.5
Total aromatics (N-free + N aromatics)	82.1	88.9	75.0	83.9	83.5
Error, %	5	7	4	5	4

## Data Availability

The authors will make the material available upon request.
